# Role of Mitochondria in Cancer Stem Cell Resistance

**DOI:** 10.3390/cells9071693

**Published:** 2020-07-15

**Authors:** José Manuel García-Heredia, Amancio Carnero

**Affiliations:** 1 Instituto de Biomedicina de Sevilla (IBIS), Hospital Universitario Virgen del Rocío, Universidad de Sevilla, Consejo Superior de Investigaciones Científicas, Avda. Manuel Siurot s/n, 41013 Seville, Spain; 2 Departamento de Bioquímica Vegetal y Biología Molecular, Facultad de Biología, Universidad de Sevilla, Avda. de la Reina Mercedes 6, 41012 Seville, Spain; 3 Centro de Investigación Biomédica en Red de Cáncer, CIBERONC, Instituto de Salud Carlos III, 28029 Madrid, Spain

**Keywords:** cancer stem cells, mitochondria, drug resistance, metabolic plasticity

## Abstract

Cancer stem cells (CSC) are associated with the mechanisms of chemoresistance to different cytotoxic drugs or radiotherapy, as well as with tumor relapse and a poor prognosis. Various studies have shown that mitochondria play a central role in these processes because of the ability of this organelle to modify cell metabolism, allowing survival and avoiding apoptosis clearance of cancer cells. Thus, the whole mitochondrial cycle, from its biogenesis to its death, either by mitophagy or by apoptosis, can be targeted by different drugs to reduce mitochondrial fitness, allowing for a restored or increased sensitivity to chemotherapeutic drugs. Once mitochondrial misbalance is induced by a specific drug in any of the processes of mitochondrial metabolism, two elements are commonly boosted: an increment in reactive nitrogen/oxygen species and, subsequently, activation of the intrinsic apoptotic pathway.

## 1. Introduction

Tumors and their microenvironment constitute a very heterogeneous structure, with multiple phenotypes that make them virtually metabolic ecosystems, different from structures in other parts of the body. These heterogeneous structures, in addition to different tumor cell types, comprise stromal cells such as infiltrated fibroblasts, endothelial cells and immune cells [1,2,3]. These non-tumor cells can greatly influence the plasticity and functionality of tumor cells [1,4]. Furthermore, within the tumor cell population, great heterogeneity exists, arising from both genetic and epigenetic differences [3,5]. Although tumors are largely clonal derivatives of a single cell, both the genomic instability of tumor cells and microenvironment variations make tumor cells a very heterogeneous population [5]. This heterogeneity allows cancer cells to adapt to different stresses that could appear in the tumor microenvironment, such as inflammation, hypoxia, low pH and nutrients. Thus, either different tumor cells or different cell metabolism properties can be found within a tumor with increasing variability, which increases the probability that a tumor can survive and invade other tissues [6,7]. 

Most tumor cells are considered differentiated—that is, unable to regenerate a tumor by themselves or metastasize—constituting the bulk of the tumor mass [8,9]. Infiltrated among them, a small percentage, generally less than 1–2% of the total, exhibits stem cell characteristics [9,10]. These cells were initially identified in leukemia (AML), but cancer stem cells (CSCs) have also been found in breast, lung, colon, brain, head and neck, prostate or liver tumors, among others [11,12]. These CSCs, also called stem-like cancer cells, tumor-initiating cells (TICs) or cancer-initiating cells, are considered to have the highest associated risk due to their ability of both self-renewal and tumor initiation in vivo, as well as their ability to invade and migrate to other tissues, leading to metastasis [13,14,15]. These cells, which have a low proliferation rate, are the only cells within a tumor capable of generating tumor cells with different characteristics, an attribute shared with stem cells. Thus, tumors may arise due to mutations that occur in normal stem cells or from differentiated cells that acquire stem-like features [16,17]. Thus, an abnormal increase in gene transcription related to stem cell regulatory pathways, such as c-MYC, Bmi-1, Hedgehog, Notch and Wnt, has been observed in CSC populations [18,19]. Similarly, stress conditions in the tumor microenvironment, typically with low oxygen levels and a limited nutrient supply, promote the epithelial-to-mesenchymal transition (EMT), leading to increased self-renewal and cell migration [7,13,20].

CSCs have been shown to be resistant to commonly used chemotherapeutic agents in leukemia, malignant melanoma, and brain, head and neck, breast, pancreas and colorectal tumors [21,22,23,24,25,26,27]. Similarly, brain and breast tumors have also shown resistance to radiation therapy [28,29]. Thus, CSCs are responsible for tumor relapse in many tumor types because they can resist cancer therapies, either intrinsically or because of changes arising from treatment. The relative abundance of CSCs is associated with the clinical outcome [30]. Thus, although traditional chemotherapy and radiotherapy can remove most tumor cells, CSCs are thought to survive, developing resistance during treatment [18,20,26,31,32]. Consequently, these cells remain hidden in the body of the patients until its reactivation [33], regenerating the tumor or migrating to other organs to metastasize. This makes them an attractive target to design new cancer therapies, because conventional treatments can kill, by autophagy and apoptosis, most of the tumor, but do not affect CSCs [13,14,28,34,35]. This fact increases the CSC population and causes a more aggressive tumor relapse [14,15,26]. The cellular mechanisms underlying this phenotype of drug resistance remain largely unknown, although there are some common elements, including the following:

—Cell quiescence, that is, a state in which CSCs do not divide, or do so very slowly [13,15,27,36,37]. Quiescence properties, such as maintenance in the G0/G1 stage in stem cells, protect them from cytotoxic stress [38]. Most chemotherapeutic drugs are based on tumor cells dividing faster than normal cells, producing little or no effect on CSCs [39]; 

—Overexpression of drug transporters that allow an active outflow of the drug [40,41,42]. Thus, the ABC genes constitute a broad family of ATP-dependent transporters in which at least 16 of them have been linked to resistance to anticancer drugs [43]. Moreover, a similar phenotype is caused by the loss of cell surface receptors or transporters that allow the drug to enter the cell [14,44]; 

—An increase in the levels of expression of the molecular targets of chemotherapeutic drugs, which causes, consequently, a decrease in effectiveness [44,45], that can be achieved, in a similar way, by the accumulation of chemotherapeutic drugs in intracellular vesicles, reducing its biological effect [46];

—Increased ability to repair DNA damage [28,42]. Treatment with chemotherapeutic drugs, such as cisplatin or carboplatin, can induce apoptotic death in sensitive cancer cells through the accumulation of DNA damage, reaching a level that makes repair impossible;

—Overexpression of antiapoptotic proteins or reduced expression of proteins related to apoptosis [47,48]. Certain CSCs exhibited higher expression levels of prosurvival proteins from the BCL-2 family [49];

—Resistance to reactive nitrogen and oxygen species (RNOS) [14,15,35,36]. Many drugs produce RNOS in excess, usually generating DNA damage and modifying protein functionality, triggering apoptosis;

—Deregulation of autophagy [13,34,42], the process in which double-membrane vesicles (autophagosomes) encompass different parts of the cell, including organelles, allowing the recycling of its components after fusion with the lysosome. This process is highly related to the maintenance of CSCs but is not restricted to them. In cancer-associated fibroblasts (CAFs), an increase in autophagy has been observed, increasing the nutrients received by tumor cells [3,4]. CAFs also provide cytokines that stimulate mitochondrial biogenesis in cancer cells [50]. Likewise, an increment in mitochondrial autophagy (mitophagy) reduces oxidative metabolism, making tumor cells dependent on glycolysis and less efficient in generating ATP and favoring a slow cell cycle, typical of some stem cells [13,14,34,35,51].

—Metabolic changes. Most CSCs exhibit high metabolic plasticity, which would facilitate the ability of these cells to thrive in adverse microenvironmental conditions, such as those of hypoxia that usually exist in tumors [6,18,35,36]. This metabolic plasticity would allow them to modify their metabolism from oxidative respiration to aerobic glycolysis. In this way, and even within the same tumor, CSCs with glycolytic and oxidative metabolism can coexist, in addition to differentiated cells with different metabolic phenotypes, because of different genetic or microenvironmental factors [52,53]. Additionally, other metabolites, such as fatty acids, ketones or amino acids, can be used by cancer cells to support metabolism [6,35,54]. Due to the crucial contribution of metabolism in malignant transformation and tumor progression, metabolic reprogramming/plasticity has become one of the characteristics of cancer.

Many of these pathways are mediated by redox imbalance and the involvement of ROS detoxification systems, which usually show CSCs involved in the upregulation of ROS metabolism [14,55]. In this way, tumor cells can modulate different metabolic pathways to obtain energy and different metabolites, while also maintaining redox balance. In many of these processes, the mitochondria play an essential role. Thus, they are responsible for the synthesis of many metabolites, participating actively in apoptotic mechanisms, in addition to being the bioenergetic power station of the cell and where most intracellular ROS are produced and eliminated [52,53]. Thus, in some CSCs, an increase was observed in the transcription of nuclear genes that encode mitochondrial proteins [56,57]. These results suggest that certain CSCs depend on both metabolism and mitochondrial biogenesis for their survival and spread. Throughout this review, we will focus on the role of mitochondria in the mechanisms of CSC resistance to different drugs, as well as in possible strategies that enable the circumvention of these mechanisms.

## 2. Mitochondria as a Crucial Element in Cancer

Mitochondria perform an essential function in cells by coordinating both the production and distribution of energy through oxidative phosphorylation (OXPHOS) based on oxygen and substrate availability. It also carries out other important metabolic reactions, such as the citric acid or tricarboxylic acid (TCA) cycle, fatty acid oxidation (FAO) or glutaminolysis. Due to its role in essential cellular function, this organelle has been linked to multiple aspects of tumorigenesis and tumor progression [58]. 

In many metabolic pathways in which mitochondria are involved, alterations have been found in tumor cells and, specifically, in CSCs [35,53,54,59,60]. Consequently, multiple chemotherapeutic drugs target mitochondria, either directly or through pathways regulating mitochondrial activity. This turns drugs that target mitochondria into promising agents to interfere with tumor adaptations, allowing the elimination of CSCs [61]. It is important to consider that mitochondria do not play a unique role in tumorigenesis and tumor progression or the response of tumor cells to treatments. Depending on the tissue in which the tumor appears, there will be epigenetic or tumor microenvironment differences that can modify mitochondrial functionality [5,54,59]. Even within the same tumor, differences between cells can mean differences in mitochondrial functionality or metabolism, thus promoting tumor adaptation to its microenvironment and the resistance capacity of the cells. 

We must consider the cell as a whole so that modifications in an organelle will have side effects in other parts of the cell. To illustrate this, we can consider the direct communication between mitochondria and the nucleus, called the retrograde response, which suggests that changes in mitochondrial physiology or metabolism may induce changes in gene expression [51,52,54,57]. Thus, the NAD^+^/NADH ratio, levels of acetyl-CoA, ATP, ROS or specific mitochondrial metabolites are involved in this process [62]. Furthermore, because most of the proteins that play a role in the mitochondria are encoded in the nucleus, there is a required nucleo-mitochondria communication to allow not only the proper mitochondrial function but also the replication of mitochondrial DNA (mtDNA) through the import into mitochondria of proteins such as TFAM, POLG and POLGY2 [52,63]. 

Multiple signaling pathways can modify mitochondrial function, such as the PI3K/AKT/mTOR pathway, with multiple roles in tumorigenesis [64]. This fact makes it challenging to describe in a review all the processes implied in CSCs’ drug resistance mediated by mitochondria. Thus, herein, we will mainly focus on mitochondrial processes. If we analyze the cycle of a mitochondrion, three key steps are involved: the generation of new mitochondria (biogenesis), mitochondrial metabolism, and the autophagy of aging or non-functional mitochondria (mitophagy) [13,34,54,60,65]. For each step, CSCs exhibit resistance mechanisms to different drugs. To consider the mitochondrial mechanisms of CSC resistance, the crosstalk between different processes inside a cell must be discussed. Multiple drugs generate oxidative stress, inducing increased ROS, against which there are multiple defense mechanisms in mitochondria [14,55]. Although some drugs cause DNA damage that will produce increased ROS, other drugs produce increased ROS that will damage DNA [66,67]. Additionally, mitochondria are crucial in cell death/survival due to the role of some of their related proteins, such as cytochrome c, in triggering the intrinsic apoptotic pathway [68]. Throughout this review, we will discuss how alterations in the regulation of each of the three key steps (biogenesis, metabolism and mitophagy) modify the resistance of CSCs to different chemotherapeutic drugs. In addition, we will discuss how that resistance can be overcome, inducing apoptotic pathway for tumor cell clearance.

## 3. Mitochondrial Biogenesis and CSC Resistance

**Figure 1:**
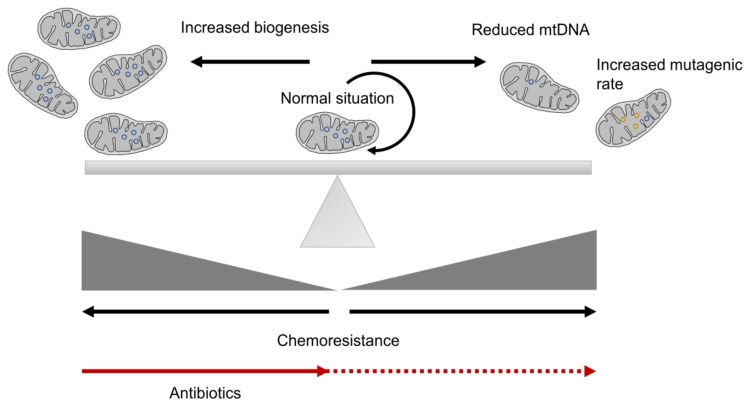
Mitochondrial biogenesis misbalance in cancer stem cells (CSCs). In normal cells, mitochondrial mass is usually maintained. However, both increased mitochondrial biogenesis, on the one hand, and alterations on mtDNA, on the other hand, are connected with an increased resistance in CSCs. Black arrows refer to increased chemoresistance, from a normal situation where mitochondrial biogenesis allows the maintenance of mitochondrial population. Treatment with antibiotics of CSCs with high mitochondrial biogenesis can overcome chemoresistance (red arrow), but prolonged treatments can force the appearance of a CSC population with reduced mtDNA (dotted red arrow).

Mitochondrial biogenesis is a crucial step in the maintenance of the cell cycle and for correct metabolism. In all cells, mitochondria must be synthesized from a pre-existing one, an essential step for correct cell division [65]. Each cell contains a variable number of copies of mitochondrial DNA (mtDNA), encoding 13 polypeptides essential for OXPHOS, as well as rRNAs and tRNAs, with the remaining mitochondrial proteins encoded in the nuclear genome [57]. Thus, mitochondrial proteins encoded by nuclear and mitochondrial genes must be transcribed in a coordinated way to form some of the mitochondrial complexes associated with OXPHOS [69]. Increased mitochondrial biogenesis is usually connected with a higher tumorigenic rate [70,71], which supports the maintenance of stem-like properties in a certain population of CSCs, independently of their metabolism [72,73,74]. Thus, an increased mitochondrial mass is usually detected in CSCs, reflecting increased mitochondrial biogenesis, which is also related to increased chemoresistance [18,72,73,75,76] (Figure 1). However, this is not a universal event because it has been also reported that cells with increased mitochondrial biogenesis are less glycolytic and exhibit reduced invasive properties [77].

According to endosymbiotic theory and the bacterial origin of mitochondria, antibiotics designed for bacteria also target mitochondria. This means that some antibiotics can be used to inhibit the spread of CSCs. Among these are doxycycline (tetracycline), tigecycline (glycylcycline), azithromycin (erythromycin), antiparasitic drugs (pyrvinium pamoate, atovaquone), and antimycobacterial drugs, such as bedaquiline [75,78]. These drugs have acceptable side effects, which would allow their use as therapeutic agents to eradicate CSCs [75] (Figure 1). Thus, doxycycline, tigecycline and azithromycin inhibit protein translation, which blocks mitochondrial biogenesis [73,75,79,80]. Due to the high number of mitochondria in many tissues, such as the skeletal muscle, brain, and heart, normal cells would be slightly affected by antibiotic treatment. However, mitochondrial biogenesis is higher in tumor cells, with a higher mitochondrial mass, making them more sensitive to these antibiotics. Clinical trials with doxycycline and azithromycin showed positive effects, such as increased patient survival and a reduction in the percentage of CSCs in multiple cancer types [81,82]. Additionally, doxycycline was shown to overcome paclitaxel resistance in CSCs [79] and reduce the CSC population in early breast cancer patients [81]; additionally, it is a strong radiosensitizer [75]. Furthermore, this drug could target paclitaxel-resistant CSCs [79]. However, continuous treatment of cancer with antibiotics may prove ineffective in the long term due to the appearance of resistance in cultured cells caused by alterations in mtDNA, such as its reduction (Figure 1). In this way, doxycycline-resistant CSCs, with lower mtDNA content, exhibited an inflexible metabolism [83]. Thus, the cells, according to glycolysis, are more sensitive to other metabolic inhibitors, such as Vitamin C, which has been described to block aerobic glycolysis by targeting glyceraldehyde-3-phosphate dehydrogenase (GAPDH) [84].

To complete correct mitochondrial biogenesis, mtDNA must be correctly maintained. The mutational rate of the mitochondrial genome is higher than that exhibited by the nuclear genome [85], with frequently detected mutations being associated with the appearance of tumors [86] (Figure 1). Thus, mtDNA damage can induce tumor progression to an advanced phenotype in different tumor types [57]. The presence of mutations in the mitochondrial genome is common in tumors, with frequent resistance to chemotherapeutic agents such as 5-fluorouracil (5-FU) and cisplatin facilitating these mutations’ resistance to other drugs, such as carboplatin, based on a similar principle of drug action [87]. Damage to mtDNA, or even its loss, causes a reduction in the rate of cell proliferation, characteristic of CSCs [39,52]. Thus, large mtDNA deletions and a reduced copy number of mtDNA by the cell are usually associated with advanced tumors, increased metastasis and a poor prognosis [52,57]. Additionally, a low mtDNA content was detected in up to 80% of breast tumors [88]. These tumors with low mtDNA content also induced EMT and an increase in invasive and metastatic properties, constituting a more malignant phenotype [89]. Furthermore, despite poor metabolism, anti-apoptotic and survival pathways are activated by retrograde signaling from the mitochondria to the nucleus [52]. Therefore, these traits produce an increase in the population of CSCs resistant to chemotherapeutic drugs, such as vincristine, doxorubicin and paclitaxel [52,57,90]. Thus, mitochondrial-deficient cells have shown resistance to increased ROS levels, such as direct treatment with hydrogen peroxide or ROS-inducing agents, such as doxorubicin, paraquat and menadione, possibly due to increased expression levels of antioxidant enzymes [91]. Similarly, mtDNA-depleted cells also showed resistance to cisplatin, hydroxytamoxifen, paclitaxel or TNF-induced apoptosis in different cancer cells [90,92,93]. However, despite the damage to mtDNA, or even its absence, the mitochondrial structure, mainly encoded in the nuclear genome, is necessary to allow the CSC phenotype. Indeed, non-tumor cells with mtDNA-deficient mitochondria can resist staurosporine-mediated apoptosis by increased expression of the antiapoptotic proteins BCL-2 and BCL-XL, sequestration of proapoptotic factors (BID, BAX, BAD) in the internal mitochondrial membrane and reduced activation of caspases 3, 8 and 9, among others properties [94]. These results show that, in general, the reduction in mtDNA content is related to increased resistance to different drugs. Additionally, mtDNA-depleted mitochondria usually show defective mitochondrial function, with a common metabolic switch towards the Warburg effect [52,86]. Interestingly, cells with low mtDNA levels exhibited lower levels of the tumor suppressor BRCA2, becoming more sensitive to PARP inhibitors, probably due to synthetic lethality [95,96].

Mitochondrial biogenesis is finely tuned by cells, with multiple pathways or regulators, such as PGC-1α, MYBBP1a or the MAPK/ERK pathway, which can modify their activity [63,97,98,99,100]. PGC-1α (peroxisome proliferator-activated receptor gamma coactivator 1-α) is an important regulator of the transcription of nuclear-encoded genes implicated in mitochondrial biogenesis [97]. Through its role as a transcriptional coactivator, PGC-1α also regulates both OXPHOS and ROS detoxifying enzymes [101]. Thus, in breast cancer, higher PGC-1α expression is correlated with a poor prognosis [102]. The use of XCT790, a compound that reduces colony formation in soft agar by regulating PGC-1α, showed a reduction in CSCs [73]. Additionally, pancreatic CSCs exhibited high levels of PGC-1α, making these cells more sensitive to metformin [18]. The MAPK/ERK pathway is also implied in mitochondrial biogenesis due to its role in both intrinsic and acquired resistance to MAPK inhibitors (MAPKi). MAPKi treatment showed the presence of a resistant population, with high mitochondrial biogenesis and an active OXPHOS metabolism [63].

All these results show that mitochondrial population should be finely tuned in normal cells to avoid an imbalance, either due to excessive biogenesis or due to defects (mutations or deletions) in mtDNA, which will probably cause changes in metabolism. 

## 4. Mitochondrial Metabolism in CSC Resistance

Importantly, each CSC subpopulation inside a tumor may exhibit a different metabolic profile to other CSCs or the tumor mass, with this heterogeneity associated with resistance to treatment. Thus, contradictory results have been obtained regarding the metabolic profile of CSCs. In some cases, they have been described as mainly glycolytic [61,103,104,105,106]; other articles have indicated that CSCs are primarily dependent on OXPHOS [18,61,76,107].

The metabolic status of CSCs is very important in drug resistance mechanisms because, when glycolytic CSCs differentiate and proliferate, a change from anaerobic to aerobic metabolism can be observed [14]. However, CSCs from different tumor types have also been described as depending on OXPHOS for most of their energy. The increased efficiency of OXPHOS-based metabolism would, in theory, allow cancer cells to use the nutrients better, allowing them to survive in nutritionally poor environments [35]. Studies have suggested that treatment-resistant CSCs are less glycolytic, indicating that specific OXPHOS inhibition could attack these cells, helping them to escape from damage in hypoxic areas of the tumor. Thus, cells under severe hypoxia become more resistant to irradiation than cells in normoxia [108]. 

At the molecular level, the typical metabolic flexibility of cancer is based on the reconnection among the different metabolic pathways, achieved through the synthesis or degradation of key proteins that allow metabolites to change pathways according to cells’ needs. The first of the described mechanisms of this metabolic remodeling is the so-called Warburg effect, in which cells use large amounts of glucose in the presence of oxygen [109]. This leads to the formation and secretion of lactate in a mechanism called aerobic glycolysis [86,109]. Although this mechanism is inefficient in energy production in the form of ATP, glucose absorption from tumor cells is usually higher, leading to a net ATP production similar to the level achieved with OXPHOS, caused by different mutations in respiratory complexes [48]. 

The low ROS levels present in CSCs could be due to events leading to metabolic reprogramming, which is essential for maintaining self-renewal and improving the antioxidant defense mechanism [14]. Thus, glycolytic CSCs are usually adapted to the typical hypoxic environment of many tumors, in which the glycolytic metabolism prevails to counteract a low mitochondrial level. Specific physiological metabolites, such as pyruvate, tetrahydrofolate, and glutamine, can function as cytotoxic agents in CSCs when administered at doses that disrupt the redox NADP^+^/NADPH ratio [110]. Thus, pyruvate accumulation mimics the blockade of the respiratory chain performed by the binding of antimycin A to cytochrome c reductase in mitochondrial complex III [110,111].

High ALDH1 (aldehyde dehydrogenase-1) expression levels are characteristic of CSCs, which are related to increased resistance to chemotherapeutic agents in sarcoma, breast and lung CSCs [112,113,114]. High ALDH expression protects against the toxic effects of RNOS production derived from chemotherapeutic treatment, so that its inhibition further raises the RNOS levels, triggering apoptosis [115]. High ALDH activity is associated with high levels in the mitochondrial mass, both in cell lines and samples derived from patients, suggesting higher mitochondrial biogenesis or retarded degradation of mitochondria. Thus, the mitochondrial biogenesis inhibitor doxycycline targets ALDH^+^ breast CSCs [116]. These results could be related to higher expression of mitochondrial ALDH isoforms [117].

The regulation among the enzymes of the glycolytic pathway, OXPHOS and TCA cycle, all involved in the synthesis of ATP and maintenance of the NAD^+^/NADH ratio, results in high metabolic plasticity in a large proportion of CSCs. The mitochondria take small molecules, such as pyruvate, fatty acids and amino acids, from catabolic reactions to obtain reduced power in the form of NADH and/or FADH_2_. These molecules allow, through their oxidation, the electron transfer in the mitochondrial respiratory chain from water to molecular oxygen.

Importantly, design strategies that target a type of metabolism can lead to tumor cells, especially CSCs, with higher metabolic plasticity, changing their metabolism to adapt to the new situation. Thus, considering combined treatment is important to short-circuit mitochondrial metabolic plasticity.

### 4.1. Role of the Mitochondrial Respiratory Chain in CSC Resistance

The mitochondrial respiratory chain, or electron transport chain (ETC), comprises four enzymatic complexes, among which complex II is completely encoded in the nuclear genome, while complexes I, III and IV are still encoded in mtDNA [86]. The higher mutation rate of mtDNA than nuclear DNA allows the appearance of certain mutations that can affect the assembly of the mitochondrial complexes but allowing its partial functioning. Thus, due to the heteroplasmy existing in cells [86], mutations in mtND1 allow the functioning of Complex I but, when the mutated population reached a threshold, they behave as tumor suppressors, preventing the assembly of complex I and also reducing its metastatic ability [118]. Thus, mtDNA mutations comprising both the activity and assembly of OXPHOS complexes may decrease or even inhibit tumor growth in xenograft models [118]. However, a partial ETC inhibition caused by mutations in mtDNA, that mimics the effect of mtDNA depletion, promoted a migratory phenotype in cultured cells, behaving as oncogenic mutations [119]. In this way, certain mutations behave as oncogenic when appearing at low levels in the mtDNA population but became tumor suppressors in homoplasmy.

**Figure 2:**
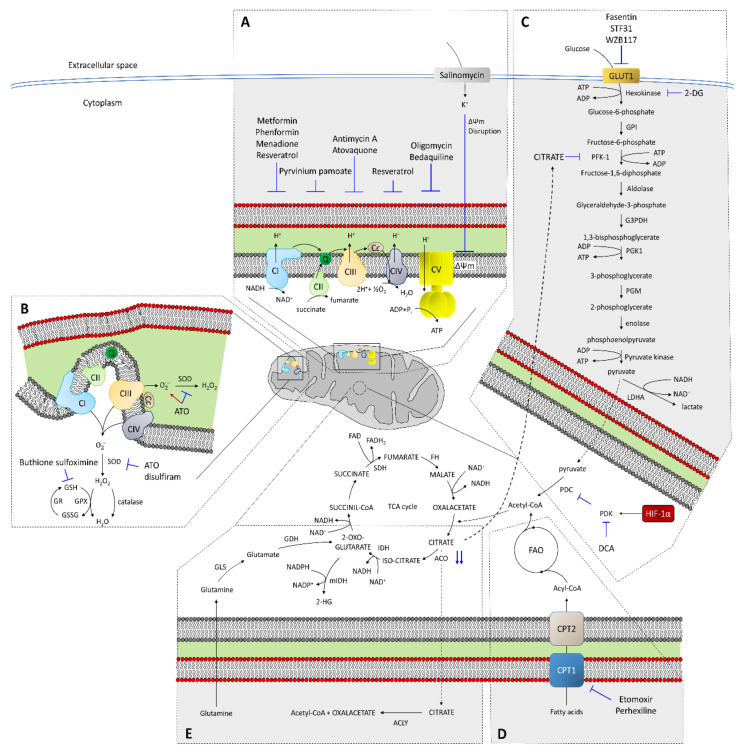
Mitochondrial metabolism targeting can overcome CSC resistance. (**A**) Mitochondrial eltron transport chain (ETC), core of oxidative metabolism, showing some of the described inhibitors for each complex (blue lines) used for CSC treatment. CI: complex I; CII: complex II; CIII: Complex III, CIV: Complex IV; CV: Complex V; Q: ubiquinone; C*c*: cytochrome c. Outer mitochondrial membrane appears in red, while inner mitochondrial membrane is colored in grey. Salinomycin causes mitochondrial membrane potential (ΔΨm) disruption. (**B**) Reactive oxygen species produced as a consequence of ETC. Inhibitors of mitochondrial detoxifying enzymes are labelled with a blue line. Red arrow indicates a RNOS enhancer. ATO: Arsenic trioxide; GSH: glutathione; GSSG: glutathione disulfide; GR: glutathione reductase; GPX: gluthathione peroxidase. (**C**) Glycolytic metabolism, showing inhibitors of GLUT1 and hexokinase, blocking glycolysis at its beginning, and dichloroacetate (DCA), that inhibits pyruvate dehydrogenase kinase (PDK) and allow the incorporation of pyruvate into the tricarboxylic acid (TCA) cycle. Blue lines correspond to glycolytic inhibitors, while red arrows belong to HIF-1α, a glycolytic enhancer. 2-DG: 2-deoxyglucose; GPI: glucose-6-phosphate isomerase; G3PDH: glyceraldehyde-3-phosphate dehydrogenase; PGK1: phosphoglycerate kinase; PGM: phosphoglycerate mutase. (**D**) Summary of fatty acid oxidation (FAO) metabolism. Etomoxir and perhexiline, inhibitors of CPT1 (blue line) diminished CSC population, by reducing mitochondrial incorporation of fatty acids. (**E**) Pathways related to the TCA cycle frequently modified in CSCs. Blue arrows correspond to aconitase (ACO) knockdown. SDH: succinate dehydrogenase; FH: fumarate hydratase; IDH: isocitrate dehydrogenase; mIDH: mutated IDH. GLS: glutaminase; GDH: glutamate dehydrogenase; IDH: isocitrate dehydrogenase; ACLY: ATP citrate lyase.

Due to the role of the four complexes involved in OXPHOS in electron flux, inhibiting a specific complex is similar to that obtained by inhibiting others (Figure 2A). Drugs inhibiting mitochondrial respiration include metformin, a complex I inhibitor that reduces tumorigenesis [120]. In non-CSCs from pancreatic cancer cells, metformin induced cell cycle arrest, while apoptosis was induced in CSCs [18,107]. This molecule can reverse the resistance to chemotherapy drugs in breast cancer cells [121]. In fact, metformin treatment specifically eliminates CD44^+^/CD24^−^ CSCs, showing positive synergy with doxorubicin and resulting in delayed tumor recurrence [122]. Additionally, metformin, also described as a PI3K/Akt/mTOR signaling inhibitor, reduced CSC resistance to temozolomide, a chemotherapy drug that causes DNA damage [123]. However, metformin resistance has also been detected in certain tumors in vivo [18], making it necessary to use combined treatments or more powerful derivatives. Thus, the combination of metformin with 2-deoxy-D-glucose (2-DG), a glycolysis inhibitor, increased the cell death percentage and reduced tumor growth in xenograft models [124]. The use of phenformin, a biguanide derivative similar to metformin and a complex I inhibitor, but with higher antineoplastic efficiency, can be used alone or combined with other drugs against CSCs [125]. Combined treatment of phenformin with gossypol, an inhibitor of ALDH and various anti-apoptotic components of the BCL-2 family, suppresses stemness and produces a significant reduction in invasion capacity and cell viability [126]. Another drug, menadione, exerts a double effect through complex I inhibition and the induction of ROS, and prevents the appearance of resistance [127]. Pirvinium pamoate, a complex II inhibitor, reduces tumorsphere formation in different cancer cell lines [75]. Similarly, antimycin A and atovaquone, both complex III inhibitors, significantly reduce CSCs [78,128]. Atovaquone target CSCs preferentially, producing an increased glycolytic rate, with no effect on normal fibroblasts [78]. ATP synthase, also called Complex V, can be inhibited by oligomycin, but its high toxicity prevents its use as a chemotherapeutic drug [76]. However, it could be used at lower concentrations combined with other drugs, such as 2-DG or niclosamide, reducing the CSC percentage in glioblastoma and ovarian and breast cancer cells [129,130,131]. Another Complex V inhibitor, bedaquiline, is a drug approved for multidrug-resistant tuberculosis [132] that targets CSCs preferentially [133]. Other drugs, such as resveratrol, can correct defects in complexes I and IV, decreasing the growth rate and invasive potential of different cancer cell lines [77,134]. This drug has been described to induce mitochondrial dysfunction, cytochrome *c* release and caspase activation in pancreatic cancer [135]. In other cases, OXPHOS must be inhibited to bypass acquired resistance to certain drugs. Thus, OXPHOS promotes resistance to cytarabine, an antimetabolite, in animal AML models, and combined treatment with OXPHOS inhibitors restores sensitivity [136].

OXPHOS can also be inhibited by changes in the mitochondrial membrane potential. Salinomycin is a K^+^ ionophore that, acting as an OXPHOS inhibitor, seems to reduce the CSC percentage both in vitro and in vivo in different types of cancer [137,138,139]. Salinomycin can kill cells resistant to different chemotherapeutic drugs, such as doxorubicin, cisplatin, gemcitabine, temozolomide, verapamil or imatinib and sensitizes radioresistant cells [139]. The toxicity of salinomycin is amplified under low oxygen and/or glucose levels, increasing RNOS levels [140]. In this situation, AMP-activated protein kinase (AMPK) is activated, triggering autophagy and allowing this drug to act as an anti-CSC molecule. Thus, the combination of AMPK agonists, such as metformin and 2-DG, with salinomycin could be used to overcome CSC resistance [139]. Salinomycin also causes mitochondrial hyperpolarization, inducing mitophagy of dysfunctional mitochondria [141]. Salinomycin can target and kill cancer cells preferentially, but not primary cells [141]. A similar effect, the preference for cancer cells, has been found for another OXPHOS inhibitor, VLX600, increasing the effect of irinotecan in xenograft models [142]. This compound also induces AMPK phosphorylation in tumor cell lines [142] and sensitizes cells to glucose starvation [143].

In most of these cases, OXPHOS inhibition causes mitochondrial imbalance, causing increased RNOS levels and/or metabolic switch to glycolysis. In solid tumors, due to the poor blood supply, both the oxygen and glucose low levels will compromise metabolic rewiring from OXPHOS to glycolysis if mitochondrial OXPHOS is inhibited. Thus, as a consequence of OXPHOS imbalance, oxidative stress due to RNOS accumulation can promote the triggering of apoptosis in CSCs based on OXPHOS metabolism.

### 4.2. RNOS in CSC Resistance

Mitochondria are the main endogenous source of RNOS, among which are free radicals (^∙^OH, RO^∙^, ROO^∙^, NO^∙^, hydroxyl, alkoxy, peroxyl and nitroxyl), superoxide (O_2_^−^) and peroxides (H_2_O_2_, RO_2_H), producing approximately 90% of all intracellular RNOS [7,14,103]. Under normal conditions, because of OXPHOS, a small percentage of these RNOS are produced, functioning as signal transduction mediators or in immune response mechanisms [144,145]. Although mitochondria are the main source of RNOS, they are also very susceptible to them, explaining their presence in these organelles of enzymes, such as superoxide dismutase (SOD), catalase, and glutathione reductase, designed to eliminate these molecules [146] (Figure 2B). However, under stressful situations, these defenses are overwhelmed, causing mitochondrial damage [147]. At the same time, oxidative damage increases; thus, damaged mitochondria produce an even greater amount of RNOS [145]. 

The imbalance between RNOS production and elimination is involved in both tumor development and progression [148,149], with RNOS behaving as an oncogenic engine. However, increased RNOS levels are common for almost all non-surgical cancer treatments, such as radio and/or chemotherapy [150]. Due to the usually high RNOS levels in cancer cells, a further increase to a critical point will trigger apoptosis [151]. Tumor cell populations, due to their heterogeneity, have different RNOS levels. Thus, bulk tumor cells tend to have a higher bioenergetic metabolism than CSCs and produce higher levels of RNOS [127]. Additionally, CSCs have a high antioxidant capacity that is critical for its self-renewal maintenance and that allows them to be resistant to drugs generating oxidative stress [14,127]. Due to this increased expression of RNOS defense genes, CSCs showed higher resistance to DNA damage after combined treatment of radio and chemotherapy [127]. Thereby, the transcription factor FOXO1 is implicated in the regulation of RNOS scavengers, such as SOD and catalase, so its high expression confers resistance to oxidative stress [152]. Additionally, gene dysfunction of members of the ABC family, four of them (ABCB6, ABCB7, ABCB8 and ABCB10) identified in mitochondria [153], produce increased RNOS levels [115]. Deficiencies in ABC transporters have been shown to increase RNOS levels, which may sensitize CSCs and overcome drug resistance [153,154]. Thus, reducing antioxidant defenses of CSCs should reduce their chemoresistance.

In antioxidant defense, glutathione plays an essential role. Thus, blocking its synthesis may allow both the removal of CSCs and reduction in tumor growth [127]. For example, buthionine sulfoximine (BSO), an inhibitor of glutathione synthesis, decreased both the clonogenicity and survival of CSCs in combination with radiotherapy, both in vitro and in vivo [127]. Arsenic trioxide increases the RNOS content and depletes both glutathione peroxidase and SOD [155], reducing the CSC population in different tumor types [35]. Similarly, disulfiram not only reduced populations with stem properties in vitro, by inhibiting SOD [156], but also reversed resistance to paclitaxel and cisplatin in triple-negative breast cancer [157]. RNOS can regulate both cell proliferation and quiescence, through MnSOD activity [158]. Thus, reduced MnSOD activity favors proliferation, while increased activity drives proliferating cells to a more quiescent state, due to the increment of H_2_O_2_ [158,159]. Thioredoxin (Trx) is also an important antioxidant protein in CSCs whose expression has been connected to docetaxel resistance in breast cancer patients [160]. One of these Trx proteins, Trx2, is localized in mitochondria [161]. Trx is highly expressed in different tumors, and it has been associated with an aggressive phenotype, with increased proliferation and reduced apoptosis [160]. Auranofin, a thioredoxin reductase inhibitor, increased the sensitivity of breast CSCs to radiation therapy [162].

On the other hand, the transcription regulator NRF2 is considered a master regulator of cellular redox homeostasis, with its regulation being very important to modify both the survival and treatment resistance capacities of CSCs [53,163]. Thus, NRF2 activation is associated with a higher mitochondrial membrane potential and the best efficiency of OXPHOS, among other effects [164]. Molecules such as brusatol or apigenin, which reduce the activation and/or protein levels of NRF2, demonstrated a reduced capacity for self-renewal, as well as increased chemosensitivity of CSCs to chemotherapeutic drugs such as Taxol or doxorubicin [165,166]. 

Both the generation of RNOS and loss of mitochondrial membrane potential trigger apoptosis. Additionally, the role of some drugs, such as aminoflavone or sulindac, is to increase RNOS levels to trigger apoptosis [55]. Other molecules, such as elesclomol and shepherdin, increase RNOS by targeting members of the heat shock protein family [167,168]. Thus, HSP90 protein is specifically expressed in mitochondria from cancer cells and is absent in normal cells [169]. This specific expression has allowed the design of gamitrinibs, small molecules that selectively target HSP90 in the mitochondria from tumors, inducing apoptosis, with negligible effects on normal cells [170].

As a summary, the treatment of tumor cells with antioxidant inhibitors will increase their sensitivity to other drugs involved in the increment of oxidative stress.

### 4.3. Glycolysis

Glycolytic metabolism is usually present in both normal stem cells and CSCs from different types of tumors. Although the glycolytic pathway, per se, is non-mitochondrial, the last product of the reaction, pyruvate, enters the mitochondria, where it is oxidized to acetyl coenzyme A (acetyl-CoA), a substrate for the TCA cycle [48]. Glycolytic intermediates are used in other reactions that allow the high proliferation rates of tumor cells to be supported. In this way, glucose-6-phosphate can be used for the pentose phosphate pathway to allow the production of NADPH or to generate ribose groups, which are necessary for nucleotide synthesis [171,172].

Glycolytic CSCs usually exhibit higher expression levels of the glucose transporter GLUT1 and subsequent changes in mitochondrial activity to favor aerobic glycolysis [106,173]. In fact, in many tumor types, the influence of both oncogenes and transcription factors related to pluripotency, such as MYC, p53, KRAS, HIF1, NANOG and OCT4, have been observed in the change from oxidative metabolism to another glycolytic [48]. The glycolytic switch increases the CSC population in breast cancer cells by reducing RNOS [104]. Thus, the inhibition of enzymes involved in aerobic glycolysis can also be used in cases in which the tumor (and/or the CSCs) is fundamentally glycolytic. Additionally, the inhibition of the GLUT1 glucose transporter, through drugs such as Fasentin, STF-31 and WZB117 (Figure 2C), reduces the tumor potential of the cells, unable to capture the glucose they require for their metabolism [52]. Consequently, combined treatment with cytotoxic agents, such as gemcitabine and 5-FU, increases the triggering of apoptosis in these cells [174].

Increased levels of hypoxia-inducible factor-1α (HIF-1α) regulate the metabolic switch from OXPHOS to glycolysis [53,86,175]. HIF-1α is required for chemotherapy resistance in CSCs from breast glioblastoma and other solid tumors [67]. Thus, HIF-1α depletion in stem cells reduces the glycolytic rate and causes cell differentiation [36]. Additionally, the metabolic change from glycolysis to mitochondrial respiration, causing the stimulation of mitochondrial function, is related to cell differentiation and loss of pluripotency [176]. The metabolic switch from OXPHOS to glycolysis is mediated by pyruvate dehydrogenase kinase (PDK1), an enzyme that is transcriptionally activated by HIF-1α [177]. PDK1 inhibits pyruvate dehydrogenase complex (PDC) by phosphorylation [53] (Figure 2C). Under this scenario, the pyruvate levels rise, and tumor cells increase lactate dehydrogenase (LDHA) expression, displacing pyruvate from the TCA cycle to lactate [53].

However, glycolysis inhibition in glycolytic CSCs reduces their tumorigenic potential. Additionally, the overexpression of FBP1, the rate-limiting enzyme in gluconeogenesis, inhibits glycolysis and reduces the formation of CSC-derived structures, like spheroids, in vitro [104,178]. Dichloroacetate (DCA) modulates glycolysis through PDK1 inhibition; thus, PDC can derive pyruvate to acetyl-CoA in the mitochondria and reducing lactate levels [179] (Figure 2C). Consequently, mitochondrial metabolism switches from glycolysis to OXPHOS, reducing cell proliferation and inhibiting tumor growth and promoting apoptosis [179]. This drug, a K^+^ ion channel modulator, preferentially targets cancer cells [180]. Thus, combined treatment of DCA with drugs targeting hypoxia produced better results in animal models [181]. Additionally, in paclitaxel-resistant cell lines, the accumulation of citric acid caused by DCA treatment could restore the sensitivity to paclitaxel [182]. Citrate inhibits phosphofructokinase-1 (PFK-1), the third enzyme in the glycolytic pathway, reducing the glycolytic rate [183]. Additionally, increased hexokinase expression is associated with a lower induction of apoptosis; thus, treatment with the glucose analog 2-DG, which can be phosphorylated by hexokinase but cannot be processed further, leads to glycolysis inhibition [143].

Thus, the treatment of glycolytic CSCs with inhibitors of this pathway reduced tumor potential and activated OXPHOS metabolism, with a typical increment in RNOS levels, increasing drug chemosensitivity.

### 4.4. Fatty Acid Oxidation in CSC Resistance

Another important consideration is fatty acid oxidation (FAO), because it can also be used by CSCs [184]. Many genes involved in fatty acid metabolism are correlated with cancer metastasis, drug resistance and relapse [60]. Some tumors exhibit a high dependence on FAO for both their survival and proliferation. FAO can produce NADPH, which reduces oxidative stress as a prosurvival event [185]. The overexpression of long-chain fatty acyl-CoA synthetases is correlated with a poor prognosis in cancer patients [186]. Thus, the overexpression of carnitine palmitoyltransferase-1 (CPT1), a step-limiting reaction in FAO, is associated with tumor progression in different tumor types [60]. Thus, the specific inhibition of CPT1 by molecules such as etomoxir or perhexiline reduced the population of breast CSCs, inhibiting their growth in vitro and reducing their tumorigenic potential in vivo [79] (Figure 2D). Additionally, etomoxir sensitized hepatocarcinoma CSCs to sorafenib [187]. Similarly, in AML patients, etomoxir also reduced CSCs and, in combination with the BCL-2 inhibitor ABT-737, significantly reduced the tumor mass [188]. Another FAO inhibitor, avocatin B, is more selective than etomoxir; thus, avocation B exerts its effect on tumor cells and CSCs without affecting hematopoietic stem cells [189].

The treatment of CSCs, in which FAO is significantly boosted, with inhibitors of the fatty acid metabolism causes a reduction in its population.

### 4.5. Other Mitochondrial Metabolism Scenarios Implied in CSC Resistance

Cancer cells, in addition to consuming excess glucose, also consume amino acids for their metabolism, especially glutamine [183]. Glutamine can also be used to produce energy in tumor cells in an oxidative phenotype that involves, among others, mitochondrial biogenesis and stimulation of amino acid degradation pathways [190]. In cancer cells, glutamine enters the TCA cycle through its conversion to α-ketoglutarate [53,54,183] (Figure 2E). The truncated TCA cycle in cancer cells, due to aconitase knockdown, results in citrate accumulation and efflux into cytosol, where it is converted into acetyl-CoA and oxalacetate by ATP citrate lyase (ACLY), allowing lipid synthesis from acetyl-CoA, and gluconeogenesis [53,54,183] (Figure 2E). ACLY inhibition by a pharmacological inhibitor or RNAi reduced both tumor growth in mouse xenografts and proliferation of tumor cells in vitro, inducing cell differentiation [191]. 

Mutations in succinate dehydrogenase (SDH) and fumarate hydratase (FH) cause an imbalance in the TCA cycle and HIF-1α stabilization [118]. Additionally, mutations of both isoforms of isocitrate dehydrogenase (IDH), cytoplasmic and mitochondrial, have been found in different cancer types [59]. Consequently, mutated enzymes reduce oxoglutarate to 2-hydroxyglutarate, which has been characterized as an oncometabolite [59], allowing increased levels of HIF-1α [192] (Figure 2E).

TCA cycle inhibition, where FAO and glutamine metabolism converge, would cause a double effect. On the one hand, it can reduce tumorigenic potential, reducing CSC population. However, on the other hand, it is possible that some cells can survive by deriving metabolites to ACLY, increasing chemoresistance. 

## 5. Horizontal Transference Connected to CSC Resistance

In addition to the process previously discussed, in which nontumor fibroblasts provide nutrients to tumor cells [1], the transfer of mitochondria and mtDNA between cells in tumors has also been detected to be related to an increase in resistance to certain treatments, such as doxorubicin or ultraviolet radiation [193]. In these cases, mitochondrial transfer occurs to the cell with damaged mitochondria, allowing aerobic respiration restoration and increased chemoresistance [193,194]. This phenomenon has been observed in both co-cultured cells and tumors during chemotherapy, by the spontaneous appearance of tunneling nanotubes that allow the transfer of mitochondria from stromal to cancer cells [194].

Additionally, CAFs secrete both lactate and ketones, promoting a reverse Warburg effect in cancer cells to stimulate mitochondrial biogenesis and inhibit apoptosis [4]. Building blocks for the anabolic needs of cancer cells, such as amino acids and nucleotides, are also secreted by CAFs [4]. These CAFs, and probably other non-tumor cells, showed the significant influence of the microenvironment on tumor cell behavior, by providing metabolites that enable favoring either the glycolytic metabolism or the OXPHOS.

## 6. CSC Resistance in Mitophagy

Altered autophagy activity is related both to appearance and tumor progression. This mechanism, which promotes adaptation to stress conditions, is induced by a wide range of anticancer drugs supporting, in many cases, the survival of CSCs and resistance to therapy [13,195]. One of the mechanisms that induce autophagy is hypoxia, typical of solid tumors, in which the oxygen levels are lower in specific areas of the tumor. Indeed, HIF-1α induction can promote pancreatic tumor cell dedifferentiation to CSCs by autophagic mechanisms [196]. Likewise, oxaliplatin treatment of colorectal tumors can induce autophagy, showing an increase in the percentage of CSCs and, consequently, greater resistance to therapy [197]. Similarly, treatment with mefloquine, which inhibits lysosome formation, can eliminate CSCs, increasing the effectiveness of chemotherapeutic agents used against colon cancer [198]. Thus, autophagy is usually a prosurvival element in tumor cells in response to chemotherapy drugs.

Thus, tumor treatment with drugs inhibiting autophagy can induce apoptosis. Thus, in enzalutamide-resistant prostate tumors, treatment with autophagy inhibitors such as chloroquine (CQ) produced sensitization of the tumor cells [199]. Conventional genotoxic treatments, such as radiation or cisplatin, cause increased DNA damage that promotes the p53 pathway, which activates autophagy regulators [200]. However, it is possible that these treatments can select those cells with high autophagy levels prior to treatment: CSCs [13]. The use of combined autophagy inhibitors and other chemotherapeutic drugs seems to be a good option to reduce the survival of resistant cells of different tumor types in culture. Thus, treatment with CQ, which blocks lysosome function, in combination with other chemotherapy drugs, such as temozolomide, doxorubicin, gemcitabine, and rapamycin analogs, significantly increases patient survival [201,202,203,204,205]. CQ also increases mitochondrial ROS, showing a connection between autophagy and mitochondria [206].

**Figure 3:**
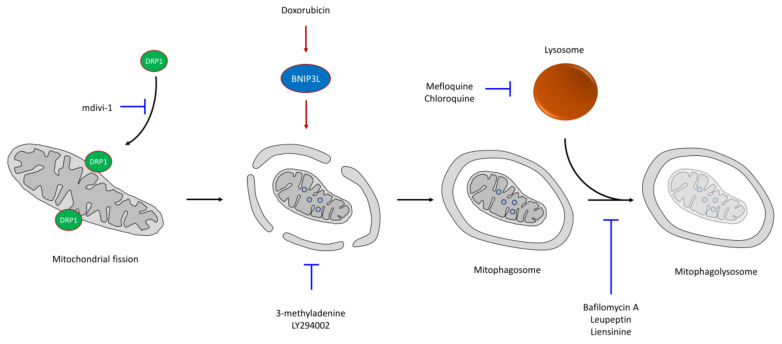
Mechanism of mitophagy. Mitochondrial fission is induced after DRP1 recruitment in mitochondria, a process that can be inhibited by targeting DRP1. Mitochondrial engulfment to form the mitophagolysosome can be inhibited in different steps. Thus, double membrane formation can be inhibited by drugs such as 3-methyladenine or LY294002, while it is enhanced by doxorubicin. Once the mitophagosome is formed, its fusion with lysosome can be inhibited by drugs like bafilomycin A, leupeptin or liensinine, or inhibiting lysosome formation by drugs like mefloquine or chloroquine. Blue lines correspond to autophagy/mitophagy inhibitors, red arrows refer to mitophagy activators.

Mitophagy—that is, selective and non-selective autophagy of mitochondria—is a mechanism that eliminates mitochondria in stressful situations, such as in the presence of moderate levels of RNOS [69,207]. During mitophagy, a double membrane is formed that engulfs the mitochondria, allowing autophagosome formation. The subsequent fusion with the lysosome, forming the mitophagolysosome, allows the recycling of metabolites upon mitochondrial degradation [34,207,208,209] (Figure 3). This mechanism also promotes the differential segregation of mitochondria, maintaining CSCs with the youngest and most functional mitochondria, while the oldest mitochondria appear in non-stem tumor cells [210]. Mitophagy, increased in the CSC population [13,198], promotes tumorigenesis and cell survival in various tumor types by allowing the removal of abnormal mitochondria. A reduction in the mitochondrial mass through mitophagy limits OXPHOS; thus, these cells rely on glycolysis, contributing to a more quiescent phenotype [36]. The opposite case, mitophagy suppression or increased mitochondrial biogenesis, produces an increase in mitochondrial respiration that promotes differentiation and reduces stemness [13]. Many chemotherapeutic agents can induce mitochondrial dysfunction and oxidative stress; thus, rapid elimination of damaged mitochondria through mitophagy is important in the resistance mechanisms to these drugs [51]. Additionally, in colorectal CSCs, increased mitophagy is associated with doxorubicin resistance by downregulating the mitochondrial apoptotic pathway and metabolic rewiring from OXPHOS to glycolysis [211]. However, importantly, excess mitophagy, which produces a deficit in the number of mitochondria in the absence of mitochondrial biogenesis, can induce metabolic disorders in the cell, promoting cell death [208]. Both esomeprazole (ESOM) and DCA are drugs capable of inducing mitophagy through increased ROS production [212,213]. 

Cationic amphiphilic drugs (CAD), including loratadine and ebastine, can exert their effect through accumulation in both mitochondria and lysosomes [214]. However, the inhibition of autophagy/mitophagy in certain steps produces increased drug sensitivity. Thus, phosphoinositol 3-kinase inhibitors, such as 3-methyladenine and LY294002, block the formation of autophagosomes, while other molecules, such as CQ, bafilomycin A1, leupeptin or liensinine, inhibit the degradation capacity of autophagolysosomes [215,216].

Due to the relationship between mitophagy and CSC resistance, the use of mitophagy inhibitors with potential antitumor drugs increases their cytotoxicity [34]. Thus, for example, the combined treatment of classic chemotherapeutic drugs, such as doxorubicin, paclitaxel, vincristine and cisplatin, together with liensinine, an alkaloid isoquinoline that inhibits mitophagy, increased the sensitivity of breast cancer cells regarding individual chemotherapy [215]. Liensinine reduces lysosome recruitment of RAB7A, which is important in the late endocytic pathway, causing the blockade of mitophagy and increased apoptosis, reducing the tumor size in a xenograft model [215]. Doxorubicin treatment increases BNIP3L expression, a regulator of mitophagy necessary for mitochondrial trapping inside autophagosomes [217]; thus, mitophagic inhibition by BNIP3L silencing increased drug sensitivity [211]. The combined treatment of doxorubicin plus liensinine induced mitochondrial fission, a mechanism associated with both mitophagy and apoptosis [53], by DRP1 accumulation in mitochondria [215]. However, the accumulation of mitophagosomes due to doxorubicin stimulation and liensinine blockage allowed apoptosis triggering [215]. The presence of higher DRP1 levels has been detected in different tumor types, increasing both mitochondrial fission and metastatic capacity [218] and conferring chemoresistance to CSCs [219]. Moreover, mitochondrial fission dependent on DRP1 is essential for stem cell maintenance due to its asymmetric mitochondrial distribution during cell division [210]. Thus, mdivi-1, which inhibits DRP1-mediated mitochondrial fission, reduced the tumorigenic potential and is a potential target for eradicate CSCs [220]. The combination of mdivi-1 with cisplatin produced a synergistic effect, causing increased ROS levels and apoptosis induction in drug-resistant cells [221]. 

Although mitophagy is associated with CSC resistance, the induction of mitophagy in specific cancer cells can also increase chemotherapy sensitivity [34]. Thus, ceramide induces DRP1-mediated mitochondrial fission and, consequently, autophagolysosomes target mitochondria, causing excessive mitophagy that is lethal to tumor cells [222]. DRP1 knockdown prevents mitophagy and inhibits ceramide localization on the mitochondrial membrane [222].

In general, the blockage of mitophagy would cause a stressful condition that increases mitophagosomes and an older non-functional mitochondria population, raising RNOS levels and, in consequence, triggering apoptosis.

## 7. CSC Resistance in Apoptosis

Apoptosis mediated by mitochondria, the intrinsic pathway, is characterized by the opening of the permeability transition pore (PTP), resulting in the release of cytochrome c, which allows apoptosome formation and subsequent activation of the caspase cascade [223]. Therefore, the inability of the cell to trigger apoptosis correctly can result in resistance to treatment [224], so mechanisms restoring this function should reverse resistance in CSCs.

**Figure 4:**
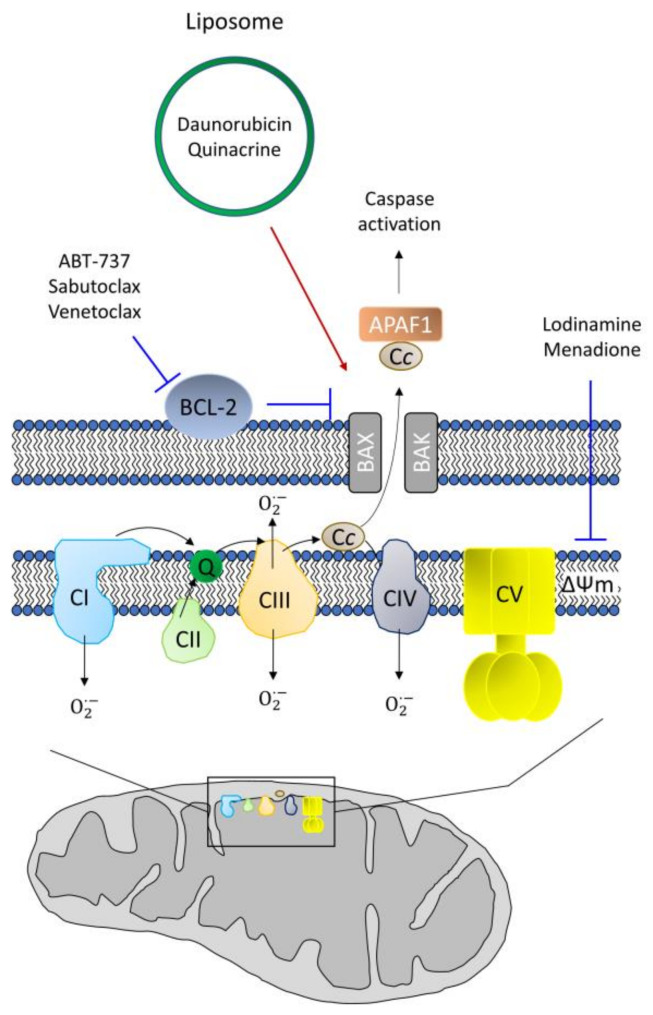
Summary of intrinsic apoptotic pathway. BCL-2, as antiapoptotic protein, inhibits the formation of pores by BAX/BAK that allow cytochrome *c* release to the cytoplasm, where it interacts with Apaf-1 to constitute apoptosome, allowing caspase cascade. Drugs targeting apoptosis comprise dissipaters of mitochondrial membrane potential (ΔΨm) or BCL-2 inhibitors (blue lines) or molecules enhancing formation of BAX/BAK pores (red arrow).

The use of drugs that cause the loss of the mitochondrial membrane potential—for example, through the permeabilization of the mitochondrial membrane—allows the induction of apoptosis. Daunorubicin- and quinacrine-containing liposomes directed against the mitochondria induced apoptosis in CSCs in vitro and murine models of tumor recurrence [225]. Similarly, lonidamine and menadione also affect the mitochondrial membrane potential, and can be used in combination with other drugs, acting as a chemosensitizing agent [226,227,228] (Figure 4).

The BCL-2 family comprises different proteins with antiapoptotic (BCL-2, BCL-XL or MCL1) or proapoptotic (BAX, BAK or BOK) properties [49]. Much of the research on the resistance to apoptosis has focused on the family members, which interact with each other [229]. The antiapoptotic members normally reside on the mitochondria, while proapoptotic members are usually localized in the cytosol, being translocated into the mitochondria to trigger apoptosis. Thus, the antiapoptotic members inhibit the function of the proapoptotic members on normal, non-damaged cells [49,229]. Additionally, mitochondrial to nuclear retrograde signaling is related to increased transcription of antiapoptotic members of the BCL-2 family and the activation of enzymes that promote survival, such as AKT [52]. Thus, a common anti-cancer strategy is the inhibition of antiapoptotic members of the BCL-2 family. The proapoptotic molecule ABT-737 binds to BCL-2 and BCL-XL, enhancing the action of BAX and BAK to act synergistically with other chemotherapeutic agents and with radiotherapy to induce apoptosis [230]. Increased levels of antiapoptotic BCL-2 family members, such as BCL-2 or BCL-XL, appear in CSCs [231] and are associated with higher resistance to both apoptosis and anticancer drugs, such as sabutoclax [232]. This drug is a small molecule that mimics the structure of the BH3 domain of BCL-2, inhibiting its antiapoptotic role. Additionally, its combination with doxorubicin produced a synergistic effect in resistant breast CSCs [232]. Another BH3-mimetic, venetoclax, allows the activation of mitochondrial proapoptotic proteins, triggering the intrinsic pathway of apoptosis [233]. However, as a common mechanism of drug resistance, tumor cell increases the expression of BCL-2 antiapoptotic proteins, conferring resistance to venetoclax in AML cells. Similar to the combined treatment of sabutoclax/doxorubicin [232], the treatment of venetoclax with tedizolid, which inhibits mitochondrial protein translation, overcomes cell resistance [234]. Paclitaxel also regulates apoptosis induction through its interaction with BCL-2, but through different resistance mechanisms, such as BCL-2 downregulation or mutation [235,236]. Resistance to this drug has also been associated with increased expression of IAPs, a family of caspase inhibitors that blocks apoptosis triggering [237]. Synergism of paclitaxel with the HSP90 inhibitor elesclomol, can help to induce apoptosis in paclitaxel-resistant cells [167]. BCL-2 inhibition also impairs OXPHOS, reducing the survival of OXPHOS-dependent CSCs in AML [238].

Due to apoptosis-acquired resistance of CSCs, the use of inhibitors of BCL2 family would reduce the CSC population.

## 8. Conclusions

Mitochondria behave as an organelle of extraordinary metabolic plasticity, capable of modifying their different pathways to support (cancer) cell survival. Additionally, the combination of different chemotherapeutic agents or radiation is a good strategy to overcome the resistance of CSCs. Thus, blocking at least two metabolic pathways simultaneously would reduce the possibility of a relapse, as well as the possible development of resistance. However, and due to metabolic plasticity, it would be important to know which pathway(s) is prevalent in a specific CSC population, in order to use drugs that could target them, increasing its effectiveness. Additionally, it would be interesting to study the design of synergistic treatments focused on CSCs mitochondrial metabolism, to reduce relapse probability.
